# Impact of SARS-CoV-2 pandemic on ophthalmological emergency visits: 1 year of experience

**DOI:** 10.5935/0004-2749.2021-0481

**Published:** 2023

**Authors:** Lucas Zago Ribeiro, Luis Filipe Nakayama, Caio Vinicius Saito Regatieri

**Affiliations:** 1 Department of Ophthalmology, Escola Paulista de Medicina, Universidade Federal de São Paulo, São Paulo, SP, Brazil

**Keywords:** SARS-CoV-2, COVID-19, Coronavirus infections, Pandemics, Emergency medical services, Ocular trauma, SARS-CoV-2, COVID-19, Infecções por coronavírus, Pandemias, Serviços médicos de emergência, Trauma ocular

## Abstract

**Purpose:**

The COVID-19 pandemic began in March 2020 and changed the healthcare system overall. The pandemic led to resource allocation changes, overloading of intensive care units, apprehensiveness of patients to seek medical care not related to COVID-19, and an abrupt reduction in all nonurgent consultations and surgeries. This study evaluated the impact on an ophthalmological emergency room for one year by assessing the correlation between societal lockdown phases and COVID-19 mortality.

**Methods:**

An observational, retrospective study was conducted that included all patients admitted to the Ophthalmology Emergency Department between January 1, 2019, and March 28, 2021. The visits were classified into prepandemic and pandemic groups that were then compared.

**Results:**

In the prepandemic period, the hospital registered a total of 71,485 visits with a mean of 194.78 ± 49.74 daily visits. In the pandemic group, there was a total of 41,791 visits with a mean of 114.18 ± 43.12 daily visits, which was a 41.4% decrease. A significant decrea­se (16.4 p<0.001) was observed in the prevalence of acute conjunctivitis, and a significant increase (6.4%; p<0.01) was observed in the prevalence of corneal foreign body disorders. A negative correlation was identified between the COVID-19 death rate and the ophthalmological inflow rates.

**Conclusion:**

This one-year analysis showed a reduction of 41.4% in emergency department visits and a significant decrease in infectious conditions. A change in hygiene habits and social distancing could explain this reduction, and the increased prevalence of trauma consultations highlighted the need for preventive and educative measures during these types of restrictive periods.

## Abstract (pt)

**Objetivos:**

A pandemia de COVID-19 foi iniciada em março de 2020 e mudou o sistema de saúde. Mudanças na alocação de recursos, sobrecarga de unidades de terapia intensiva, apreensão dos pacientes em procurar atendimento médico não relacionado ao COVID-19 e redução abrupta de todas as consultas e cirurgias não urgentes. Este estudo avalia o impacto em um pronto-socorro oftalmológico após 1 ano de pandemia, avaliando a correlação entre as fases de lockdown, a mortalidade do COVID-19 e as visitas ao pronto-socorro.

**Métodos:**

Estudo observacional retrospectivo que incluiu todos os pacientes admitidos no serviço de emergência oftalmológica do Hospital São Paulo, vinculado a UNIFESP/EPM, entre 1º de janeiro de 2019 e 28 de março de 2021. As visitas foram classificadas e comparadas em um grupo pré-pandemia e pandemia.

**Resultados:**

No período pré-pandemia, o hospital registrou um total de 71.485 atendimentos com média de 194,78 ± 49,74 atendimentos diários, e no grupo pandemia, um total de 41.791 com média de 114,18 ± 43,12 atendimentos diários, redução de 41,4%. Uma diminuição significativa de 16,4% (p<0,001) foi observada na prevalência de conjuntivite aguda e um aumento significativo de 6,4% (p<0,01) na prevalência de corpo estranho da córnea. Foi identificada uma correlação negativa entre a taxa de mortalidade do COVID-19 e as taxas de visita ao pronto-socorro.

**Conclusão:**

Esta análise de um ano mostrou uma redução de 41,4% nas visitas ao pronto-socorro, e uma diminuição significativa nas conjuntivites agudas. A mudança nos hábitos de higiene e o distanciamento social poderiam explicar essa redução, e o aumento da prevalência de traumas corneanos. Achados destacam a necessidade de medidas preventivas e educativas durante os períodos restritivos.

## INTRODUCTION

The first cluster of SARS-CoV-2 infection cases was reported in December 2019 in Wuhan, China, and a world outbreak was declared in March 2020 by the World Health Organization^(1)^.

The first COVID19 Brazilian case was reported on February 26, 2020, and more than 18 million COVID-19 cases have been reported since then^(2)^. Similar to most countries, the Brazilian government implemented restrictive social distancing measures to reduce the SARS-CoV-2 spread.

There was a great impact on the healthcare system, such as changes in resource allocation, overload of intensive care units, apprehensiveness of patients to seek medical care not related to COVID-19, and an abrupt reduction of all nonurgent consultations and surgeries.

In the Federal University of São Paulo (UNIFESP) Hospital, a public state-funded 24/7 service in São Paulo, Brazil, the emergency room (ER) remained fully open during all pandemic phases. However, all elective surgeries and consultations were postponed and then partially restarted after June 2020.

Previous studies reported significant changes in the number of ophthalmology emergency visits in different countries, such as Italy^(2,3)^, Canada^(4)^, and Israel^(5)^. However, there is a lack of data on long-term follow-up of the COVID-19 impact on health care, especially in countries that still face dramatic COVID-19 infection rates in 2021, such as Brazil.

UNIFESP’s previous analysis as of July 31, 2020, indicated there was a 58.2% reduction in patients seeking ER services and an important decrease in acute conjunctivitis diagnoses, which went from 26.6% to 13%^(6)^.

This study aimed to evaluate the impact of the COVID-19 pandemic on ophthalmological emergency sector visits one year after the onset of the pandemic and to compare the differences between partial and full lockdown stages. We analyzed the correlation between ophthalmological emergency visits and COVID-19 mortality rate.

## METHODS

An observational, retrospective study was conducted that included all patients admitted to the Ophthalmology Emergency Department of Hospital São Paulo between January 1, 2019, and March 28, 2021.

Patient demographic information (age, gender, date, and hour of visit) and International Classification of Diseases 10 (ICD-10) diagnoses were collected from medical records and hospital databases.

The visits were classified into two groups for analysis: visits from March 16, 2019, to March 16, 2020, as the prepandemic group, and from March 17, 2020, to March 17, 2021, as the pandemic period group. Additionally, a correlation analysis between the visit number and lockdown stages was performed.

Patients with missing medical information in their charts were excluded from the analysis. This study was approved by the UNIFESP ethics committee and followed the Declaration of Helsinki tenants.

The statistical analyses were performed using SPSS software (Version 25, IBM Corporation, NY, USA). An independent *t*-test was used for continuous variables and Chi-squared tests were used for categorical variables. P values of <0.05 were considered significant.

## RESULTS

During the pandemic (March 17, 2020, to March 17, 2021), the hospital registered a total of 41,791 visits with a mean of 114.18 ± 43.12 visits per day (range 27-214), and in the prepandemic group (March 16, 2019, to March 16, 2020), there were 71,485 visits with a mean of 194.78 ± 49.74 visits per day (range 71-307). The total visit number decreased by 41.4% from the prepandemic to the pandemic period, and the month with the lowest number of patient visits was in April 2020, with a mean of 59.03 visits per day.

The mean patient’s age was 41.25 ± 20.01 years in the prepandemic group and 42.76 ± 19.02 years in the pandemic group. No significant difference was observed when comparing different age groups. Male patients represented 55.3% (39,560) in the prepandemic group and 57.3% (23,938) in the pandemic group.

The day of the week with the highest inflow was Monday in both groups, representing 18.8% in the prepandemic group and 17.4% in the pandemic group. Visits between 7 am and 5 pm represented 74.3% of the visits in the prepandemic group and 75% in the pandemic group. There were no significant changes in visits according to the time of day or day of the week between the groups.

The leading diagnoses in the 2020 comparison period were acute conjunctivitis (26.6%), blepharitis (15.5%), and corneal foreign bodies (7.7%). In the pandemic period, the leading diagnoses were corneal foreign bodies (14.1%), blepharitis (11.3%), and acute conjunctivitis (10.25%). A significant decrease (16.4%; p<0.001) was observed in the prevalence of acute conjunctivitis, and a significant increase (6.4%; p<0.01) was observed in the prevalence of corneal foreign bodies (Table 1).

**Table 1:** Leading diagnosis in the emergency department between January 1, 2020 to March 17, 2021

	**2020 Prepandemic** January 1, 2020, to March 16, 2020	**1 Year Pandemic Period** March 17, 2020, to March 17, 2021		**2021 Pandemic Period** January 1, 2021, to March 17, 2021	
	**Visits per day (% of total)**	**Visits per day (% of total)**	**Difference**	**Visits per day (% of total)**	**Difference**
Acute conjunctivitis	33.96 (26.64)	7.76 (10.25)	-16.39%	9.72 (10.79)	-15.85%
Blepharitis	19.80 (15.53)	8.58 (11.34)	-4.20%	11.47 (12.73)	-2.81%
Keratitis	9.36 (7.34)	6.39 (8.44)	1.10%	7.96 (8.83)	1.49%
Corneal foreign body	9.88 (7.75)	10.70 (14.14)	6.39%	11.44 (12.70)	4.94%
Hordeolum/chalazion	9.25 (7.26)	3.78 (4.99)	-2.26%	4.89 (5.43)	-1.83%
Corneal ucer	6.00 (4.71)	4.38 (5.79)	1.08%	4.51 (5.00)	0.29%
Ocular trauma	5.08 (3.98)	4.70 (6.21)	2.23%	5.77 (6.41)	2.42%
Subconjunctival hemorrhage	4.34 (3.41)	2.49 (3.30)	-0.11%	3.19 (3.54)	0.13%
Cataract	2.49 (1.95)	1.57 (2.08)	0.12%	1.73 (1.92)	-0.03%
Uveitis	3.66 (2.87)	3.67 (4.85)	1.98%	3.24 (3.60)	0.73%
Glaucoma	2.57 (2.01)	2.71 (3.58)	1.57%	2.53 (2.81)	0.80%
Retinal detachment	2.14 (1.68)	1.86 (2.45)	0.77%	1.83 (2.03)	0.34%
Retinal vascular alteration	2.30 (1.81)	2.41 (3.18)	1.38%	3.80 (4.22)	2.41%
Corneal graft complications	1.25 (0.98)	1.29 (1.70)	0.72%	1.63 (1.81)	0.82%
Chemical eye burns	1.03 (0.81)	1.57 (2.07)	1.26%	1.97 (2.19)	1.38%

During the first lockdown phase, the emergency visits abruptly reduced, and the number of deaths increased. Even before the flexible period, the deaths related to COVID-19 progressively decreased and the visits increased until November 2, 2020. In the next phase, the deaths increased, and emergency visits progressively decreased.

## DISCUSSION

**Figure 1:**
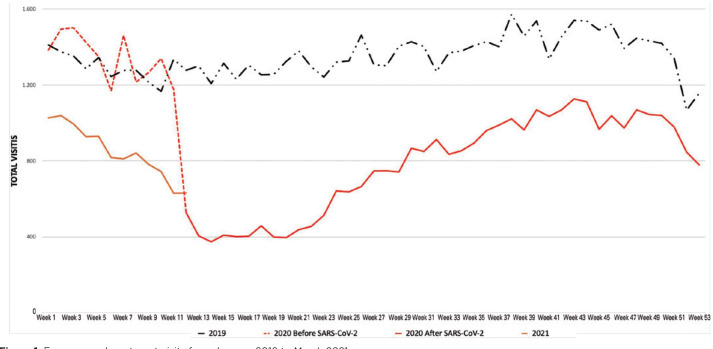
Emergency department visits from January 2019 to March 2021.

The COVID-19 pandemic has had a great impact on the healthcare system overall. The number of people seeking emergency care has decreased. Our group reported a decrease of 58.2% in the first four months of the COVID-19 pandemic^(6)^, and in this one-year analysis, a reduction of 41.4% in visits to UNIFESP ophthalmological ER was recorded (Figure 1).

**Figure 2:**
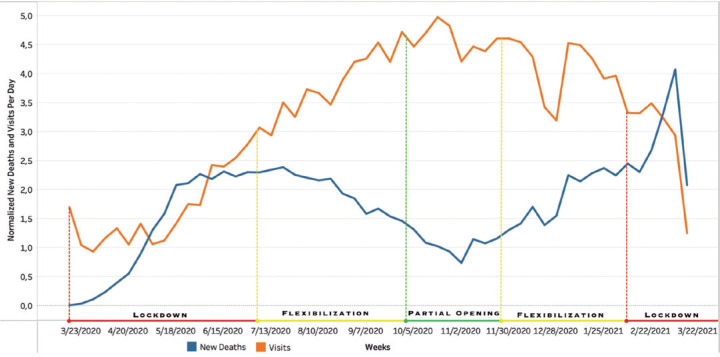
Normalized emergency department visits per day and new deaths per day from COVID-19 in Brazil from March 17, 2020, to March 17, 2021. The periods of quarantine phases in São Paulo, Brazil, were compared.

A negative correlation was found between the Brazilian COVID-19 death rate and the ophthalmological ER inflow rates^(7)^. In São Paulo, the government defined four degrees of quarantine: the red phase (lockdown with the highest restrictive measures), the orange phase (lockdown with some restrictive measures), the yellow phase (partial flexibilization), and the green phase (partial opening; Figure 2).

Previous studies reported changes in the first months of the pandemic in ophthalmologic ER centers with less than 50 visits per day. Pellegrini et al. found a reduction of 73% in visits to an Italian ER until April 2020^(3)^, and Posarelli et al. found a decrease of 65.4% until May 2020^(2)^. Yehezkeli et al. found a decrease of 43% until April 2020 in a center in Israel^(5)^. Most studies did not find any significant changes related to age, gender, or time of visit to the ER, which was similar to our findings.

This one-year analysis contributed to a better understanding of the real impact of the pandemic in the long term and can guide ophthalmology management in similar future situations to reduce unnecessary vision loss. Our study showed that the impact on ophthalmological access due to the pandemic persisted even after one year. We are still facing “new waves” of high death rates in the first semester of 2021, which is a different reality from other countries that could reduce the impact of a pandemic faster with effective vaccines and positive case monitoring programs. Development of a teleconsultation program^(8)^ and better-defined workflows^(9)^ for orienting patients and ophthalmologists are good lessons that could be gained from the pandemic.

The COVID-19 pandemic led to unprecedented changes in hygiene habits and social distancing. Patients presenting with acute viral conjunctivitis, which could be a rare related COVID symptom^(10)^, has significantly dropped from 26.6% to 10.2%. Compared to our previous analysis of ER visits until July 2020, which showed a prevalence of 12.9% for conjunctivitis, the updated data showed that it continued reducing until March 2021. In contrast, an increased prevalence of traumas and corneal foreign bodies was reported and this highlighted the need for preventive and educative measures during the restrictive periods.

There is no previous period described in the literature that has led to this level of reduction in acute conjunctivitis, and the data were similar to our daily experience in São Paulo, Brazil. Will acute conjunctivitis abruptly increase after completely stopping social distancing and pandemic hygiene habits? There are no studies that have answered this question, even in countries that have already reduced restrictions due to high vaccine coverage.
